# Transcriptomics and metabolomics reveal effect of arbuscular mycorrhizal fungi on growth and development of apple plants

**DOI:** 10.3389/fpls.2022.1052464

**Published:** 2022-10-25

**Authors:** Shan Jing, Yuchao Li, Lingcheng Zhu, Jing Su, Tianyi Yang, Bowen Liu, Baiquan Ma, Fengwang Ma, Mingjun Li, Manrang Zhang

**Affiliations:** State Key Laboratory of Crop Stress Biology for Arid Areas, College of Horticulture, Shaanxi Key Laboratory of Apple, Northwest A&F University, Yangling, Shaanxi, China

**Keywords:** *Malus × domestica*, mycorrhizal symbiosis, transcriptome, metabolome, root-associated fungus

## Abstract

Arbuscular mycorrhizal fungi (AMF) and plants form a symbiotic relationship that promotes plant growth and development. However, the regulatory mechanisms through which AMF promote plant growth and development are largely unexplored. In this study, the apple rootstock M26 was assessed physiologically, transcriptionally and metabolically when grown with and without AMF inoculation. AMF significantly promoted the number of lateral root (LR) increase and shoot elongation. Root transcriptomic and metabolic data showed that AMF promoted lateral root development mainly by affecting glucose metabolism, fatty acid metabolism, and hormone metabolism. Shoot transcriptomic and metabolic data showed that AMF promoted shoot elongation mainly by affecting hormone metabolism and the expression of genes associated with cell morphogenesis. To investigate whether shoot elongation is caused by root development, we analyzed the root/shoot dry weight ratio. There was a correlation between shoot growth and root development, but analysis of root and shoot metabolites showed that the regulation of AMF on plant shoot metabolites is independent of root growth. Our study bridged the gap in the field of growth and development related to AMF.

## Introduction

Arbuscular mycorrhizal fungi (AMF) are widely distributed in the ecosystem. Symbiosis with arbuscular mycorrhizal (AM) from the Order Glomales is observed in 80% to 90% of terrestrial plants worldwide (Gadkar et al., 2001). AMF can improve plant resistance to biotic stressors, such as insect pests and bacterial and fungal diseases, and abiotic stressors, such as drought, salt, and heavy metals (Karthikeyan et al., 2016; Gao et al., 2020; Huang et al., 2020; Adeyemi et al., 2021). In addition, AMF promote the growth of plants, including perennial trees like apple, trifoliate orange, and catalpa (Hosseini and Gharghani, 2015; Liu et al., 2018; Chen et al., 2020). The mechanism through which AMF improves plant stress tolerance has been extensively studied, but there is not yet intensive study on how AMF regulates plant growth and development.

AMF is a specialized, obligate biotrophic fungus that must inhabit a plant root. Once established, this symbiosis can promote root development (Hosseini and Gharghani, 2015; Liu et al., 2018; Huang et al., 2020). It remains ambiguous how AMF promotes root development in plants. Numerous researchers have proposed that AMF symbiosis promotes the uptake of mineral nutrients, such as phosphate and nitrogen, which would then promote root development (Bonfante and Genre, 2010; Andrino et al., 2021). Wang et al. (2021) concluded that auxin plays a critical role in AMF promotion of plant growth. Although it is commonly understood that AMF promote root development, the molecular mechanisms underlying this phenotype are still poorly studied.

As the same time, AMF also facilitate the development of the shoot. In apple and tomato, inoculated plants have a greater plant height, stem diameter and dry shoot weight (Gao et al., 2020; Huang et al., 2020; Wang et al., 2021). In a two-year field experiment, AMF promoted the plant height of *Helianthus tuberosus* in a comparison between mycorrhizal and non-mycorrhizal plants (Nacoon et al., 2021). AMF also affects the hormone content in stem, including the levels of IAA, cytokinins (CTK), and gibberellic acid (GA) (Dugassa et al., 1996; Barker and Tagu, 2000). It has long been believed that the growth potential of the above ground parts of the plant is improved through the increased root development. However, it has not been confirmed if the promotion of shoot growth is due to better root development or due to the AMF.

Apples (*Malus* × *domestica* Borkh.) are among the most valuable horticultural fruit crops cultivated and is of great interest to horticultural researchers all over the world (Wang et al., 2018). In apple, AMF improves the resistance to stress and promotes plant growth and development (Huang et al., 2020; Gao et al., 2020). Metabolomics is a widely used and highly recognized analytical method for studying plant growth and development (Fàbregas et al., 2018; Yang et al., 2022). This study is an association analysis of metabolomic and transcriptomic datasets between the roots and shoots of apple plants before and after inoculation with AMF. Based on our analysis, we provide a partial explanation of how AMF regulates root and shoot growth and development at both the metabolic and transcriptional levels. We found that the promotion of shoot growth by AMF was not merely due to better root development, as AMF also directly regulated shoot growth and development. These results may provide a new direction for the study of AMF regulation of plant growth and development, that AMF may directly affect shoot development through some pathways, such as effectors, RNAs or metabolites of AMF.

## Results

### Plant growth and physiological responses to inoculation with AMF

**Figure 1:**
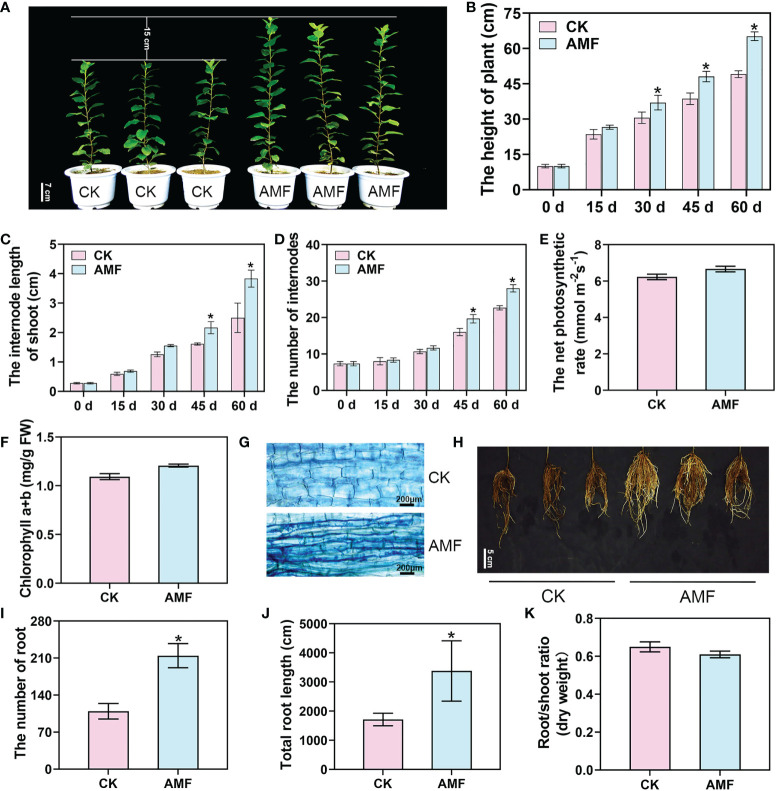
Apple plant growth and physiological responses to AM symbiosis. **(A)** The phenotype of mycorrhizal and non-mycorrhizal apple plants inoculated with *Rhizophagus irregularis* for 60 days. Scale bar = 7 cm. CK, non-mycorrhizal inoculated plants. AMF, mycorrhizal-inoculated plants. **(B–D)** The plant height, internode length, and internode number of apple plants uninoculated or inoculated with *R*. *irregularis* after 15, 30, 45 and 60 days, respectively. **(E, F)** The net photosynthetic rate and chlorophyll content of *R*. *irregularis* mycorrhizae uninoculated or inoculated apple plants after 60 days. **(G)** Staining of mycorrhizae in apple plants uninoculated or inoculated with *R*. *irregularis* after 60 days. **(H)** The root phenotype of uninoculated or inoculated apple plants after 60 days. Scale bar = 5 cm. **(I-K)** The number of roots, total root length, and root/shoot ratio of apple plants uninoculated or inoculated with *R*. *irregularis* after 60 days. Values are the means ± SD of three biological replicates. * Indicates a significant difference at *P* < 0.05 level.

To explore the effects of AMF on plant growth and development, we inoculated young plants of the cultivar M.26 with AMF. After 30 days, the average height of the plants inoculated with the mycorrhizae was higher than that of the uninoculated plants (**Figures 1A, B**). Sixty days after inoculating with AMF, the average height of the inoculated plants was 65.2 cm, while that of the uninoculated plants was 49.1 cm, and the height of the plants was markedly different. Moreover, the internode length of the mycorrhizal-inoculated plants was 1.4 times that of the uninoculated plants and the number of internodes was 1.28 times that of the uninoculated plants (**Figures 1C, D**). Interestingly, we found that there were no significant differences in the chlorophyll contents and photosynthetic rates between the mycorrhizal plants and non-mycorrhizal plants (**Figures 1E, F**). At the same time, a good symbiotic relationship between the AMF and the plants was observed 60 days after inoculation (**Figure 1G**). Furthermore, the number of roots in mycorrhizal plants was 215, while that in the uninoculated plants was 109, and the number of inoculated roots was significantly increased 60 days of inoculation with AMF (**Figure 1H**). The total root length was 3379.9 cm in mycorrhizal plants and 1713.8 cm in uninoculated plants (**Figures 1I, J**). There was no significant difference in the root/shoot (dry weight) ratio between mycorrhizal and non-mycorrhizal plants (**Figure 1K**). These results showed that AMF promoted shoot growth and LR development.

### Overall analysis of metabolomics in root and shoot of apple plants with AM symbiosis

The metabolome is closely related to plant growth and development. To explore whether AMF affected the accumulation of metabolites, we analyzed the metabolome in the root and shoot. The quality control showed that the total ion ground curve of metabolite detection had a high overlap, indicating that the signal stability of the mass spectrometry was good (**Supplemental Figures 1A–D**). **Supplemental Figure 1E** shows that the intra-group repeatability was good. Principal component analysis (PCA) revealed that components 1 and 2 explained 77.58% and 11.44% of the variability, respectively, indicating significant metabolic diversity between roots and shoots (**Supplemental Figure 1F**).

After filtering by signal-to-noise ratio, 814 metabolites were detected in the root, with 48 differential metabolites between uninoculated and AM-inoculated roots. Among the differential metabolites, 15 were significantly decreased and 33 were significantly increased (**Supplemental Figure 1G**). In shoots, 1142 metabolites were detected, of which 173 metabolites were significantly down-regulated and 63 metabolites were significantly up-regulated (DESeq2 analysis with a |log 2(fold change)|≥1 and Variable Importance in Projection (VIP) ≥1) (**Supplemental Figure 1H**). The 824 identified metabolites in the roots included 133 lipids, 66 organic acids, 59 saccharides and alcohols, 43 nucleotides and derivatives, 59 amino acids and derivatives, 62 terpenoids, 46 alkaloids, 114 flavonoids, 37 lignans and coumarins, 13 tannins, 139 phenolic acids, 11 quinones, and 32 other compounds that did not fit into these main classes (**Supplemental Data 1**). The 1142 identified metabolites of shoot included 137 lipids, 89 organic acids, 53 nucleotides and derivatives, 85 amino acids and derivatives, 73 terpenoids, 61 alkaloids, 255 flavonoids, 31 lignans and coumarins, 14 tannins, 208 phenolic acids, 73 saccharides and alcohols, and 63 other compounds (**Supplemental Data 2**).

### Analysis of differential metabolites in apple roots and shoots in symbiosis with AM

**Figure 2:**
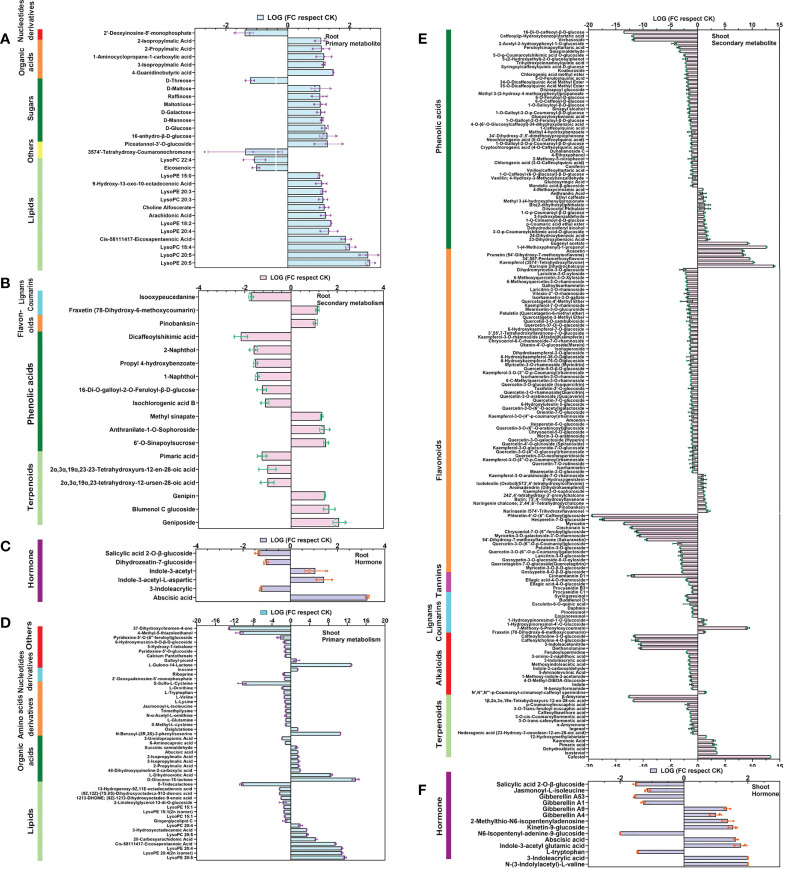
Heatmap of differential metabolites in roots and shoots of apple plants inoculated with *R*. *irregularis* after 60 days, with the baseline set as the level in uninoculated plants. **(A)** Thirty differential primary metabolites were identified in roots of apple plants. **(B)** Eighteen differential secondary metabolites were identified in roots of apple plants. **(C)** Six differential hormonal metabolites in roots of apple plants. **(D)** Fifty-one differential primary metabolites in shoots of apple plants. **(E)** One hundred eight-five differential secondary metabolites in shoots of apple plants. **(F)** Fourteen differential hormonal metabolites in shoots of apple plants. Primary and secondary metabolites are shown as the ratio of relative content, while hormone metabolites were the ratio of absolute content. In the case of each hormonal metabolite, the metabolites were only detected in either the uninoculated or inoculated plant, but not in both sample sets, which was considered to be significantly regulated (|log 2 FC| = 2).

In the AM-inoculated roots, there were 48 differential metabolites, among which there were 30 primary metabolites (14 lipids, 8 sugars, 5 organic acids, 2 others, and 1 nucleotide) and 18 secondary metabolites (9 phenolic acids, 6 terpenoids, 2 lignans and coumarins, and 1 flavonoid). Among these differential metabolites, lipids, sugars, organic acids, terpenoids, and phenolic acids were visibly altered with AM symbiosis, accounting for 87.5% of all differential metabolites. Lipid-containing substances were the most abundant, with two (eicosenoic acid and LysoPC 22:4) down-regulated, while the other 12 were up-regulated (**Figure 2A**). Changes in the phenolic acids and sugars were comparatively significant, accounting for 18.8% and 16.7% of differential metabolites, respectively. Many organic acids and terpenoids were also changed (**Figure 2B**). We also measured the levels of hormones in the roots. The contents of abscisic acid, indole-3-acetyl-L-aspartic acid, and Indole-3-acetyl glycine were increased over 1-fold, while 3-Indoleacrylic acid, dihydrozeatin-7-glucoside, and salicylic acid 2-O-β-glucoside were decreased 61.8% (**Figure 2C**). These results suggested that AMF might affect the accumulation of lipids, sugars, organic acids, terpenoids, phenolic acids, and hormones in plant roots.

In the apple shoots, there were 236 differential metabolites (**Figures 2E, F**), among which 51 were primary metabolites (17 lipids, 12 amino acids and derivatives, 10 organic acid, 9 others, and 3 nucleotides and derivatives) and 185 were secondary metabolites (82 flavonoids, 57 phenolic acids, 16 terpenoids, 15 alkaloids, 10 lignans and coumarins, and 5 tannins). There were three times more differential secondary metabolites than differential primary metabolites. Significant changes were seen for flavonoids, phenolic acids and lipids, accounting for 34.7%, 24.2%, and 9.1% of the differential metabolites, respectively. We also found 17 metabolites that showed specific accumulation in inoculated plants (5 flavonoids, 4 lipids, 2 phenolic acids, 2 organic acid, 1 terpenoids, 1 lignans and coumarins, 1 amino acid derivative, and 1 others), conversely, 21 metabolites that showed specific accumulation in the non-inoculated plants (7 flavonoids, 4 alkaloids, 3 phenolic acids, 2 organic acid, 2 others, 1 amino acid derivative, 1 tannin, and 1 lipids) (**Figures 2D, E**). These data collectively indicated that the most accumulated metabolites were flavonoids, phenolic acids, and lipids. Again, there were significant changes in the roots in hormones, such as auxin, cytokinin (CTK) and gibberellin (GA), accounting for 78.6% of the total differential hormones. Of the 14 differential hormone metabolites, four metabolites were related to GA, four to auxin, three to CTKs and three to other hormones (**Figure 2F**). Altogether, our results indicated that symbiosis with AMF might affect the accumulation of flavonoids, phenolic acids, lipids, and hormones in the shoot.

### Analysis of transcripts by RNA-seq in apple roots and shoots in symbiosis with AM

In order to investigate whether metabolic pathways were transcriptionally regulated in apple inoculated with AMF, we subjected the RNA extracted from inoculated and uninoculated roots and shoots to RNA-sequencing. The intra-group repeatability was good, ranging from 0.906 to 0.999 (**Supplemental Figure 2A**). The PCA showed that components 1 and 2 were 91.7% and 5.6% of the variability, respectively, indicating significant transcriptional diversity between roots and shoots (**Supplemental Figure 2B**). There were 2371 differently expressed genes (DEGs) in root (1140 were up-regulated and 1231 were down-regulated; |log 2 FC| > 1; P value < 0.05; **Supplemental Figure 2C**; **Supplemental Data 3**) and 1142 DEGs in shoots (319 up-regulated and 823 down-regulated; |log 2 FC| > 1; **Supplemental Figure 2D**; **Supplemental Data 3**). To verify the accuracy of the transcriptome results, we randomly selected 4 DEGs (*ARF19*, *PRP1*, *IAA17*, and *YUCCA6*) for RT-qPCR using RNA from roots and shoots. The results showed that the trends for the relative expression of genes were consistent between the RNA-seq and the RT-qPCR (**Supplemental Figures 2E, F**).

### Analysis of differentially expressed genes in roots and shoots of apple plants with AM symbiosis

**Figure 3:**
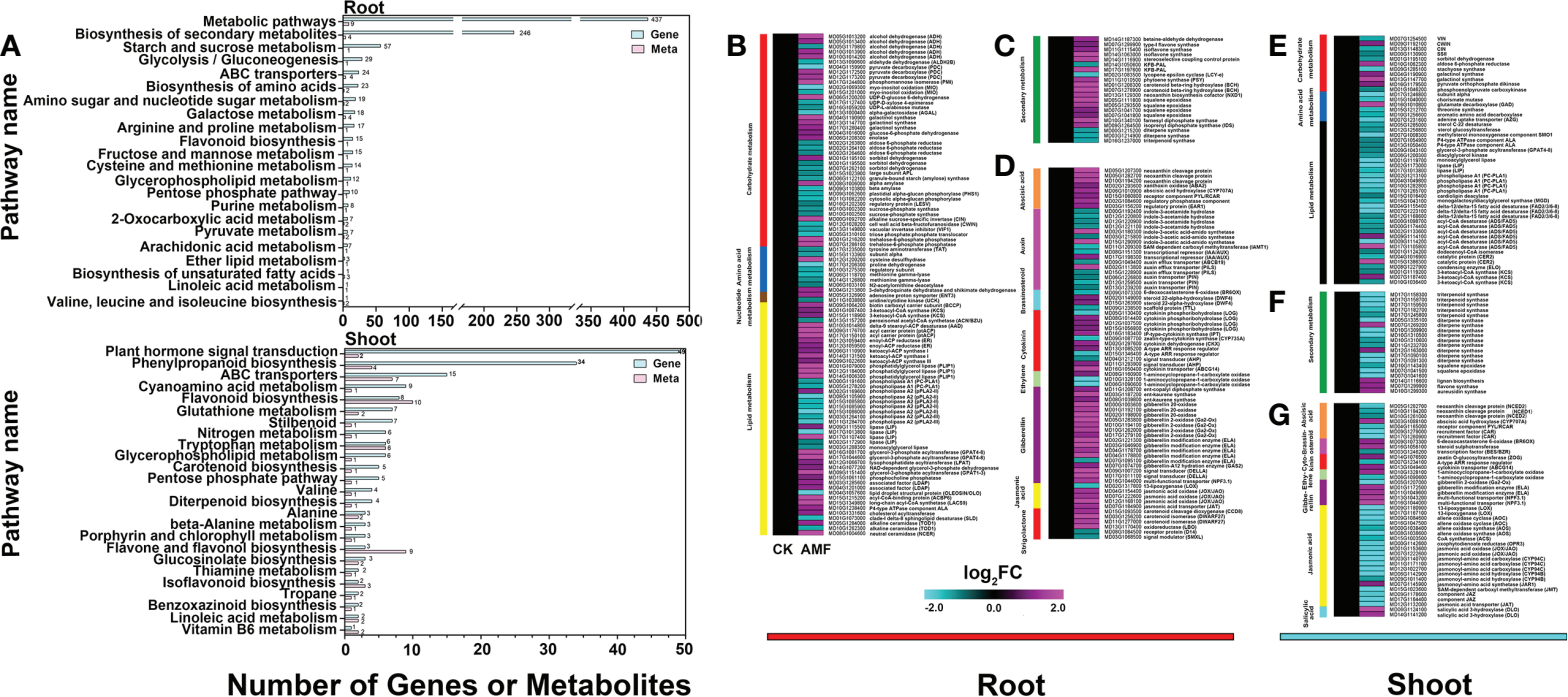
DEGs in root and shoot of apple plants inoculated with *R*. *irregularis* for 60 days (according to differential metabolites and MapMan). **(A)** Enrichment analysis of related differential genes and differential metabolites in root and shoot of transcriptome and metabolome. **(B–D)**, Differential genes related to metabolites in root, **(B–D)** with ninety-nine differential genes related to primary metabolites, twenty-one differential genes related to secondary metabolites, and seventy-three differential genes related to hormonal metabolites in root, respectively. **(E–G)**, Differential genes related to metabolites in shoot, **(E)** with forty-nine differential genes related to primary metabolites, **(F)** twenty differential genes related to secondary metabolites, and **(G)** forty-two differential genes related to hormonal metabolites in shoot. Expression-fold changes of DEGs (|log 2 FC| > 1, FDR < 0.05) were represented by color gradient from pink (induced) to blue (reduced).

The differential metabolites and the differentially expressed genes identified from the mycorrhizal-inoculated and uninoculated plants were combined for an enrichment analysis. The differential metabolites and the differential genes were enriched in the same pathways, thereby indicating that the transcriptome and metabolome corresponded (**Figure 3A** and **Supplemental Data 4**). And the datasets were next analyzed using MapMan. In roots, of the 120 differential genes, 99 genes were related to primary metabolites and 21 genes were related to secondary metabolites. Upon further analysis, the most changed genes were related to lipid metabolism (46) and carbohydrate metabolism (42). Of the genes related to lipid metabolism, 23 genes were related to glycerolipid metabolism, 12 genes were related to fatty acid metabolism, and 11 genes were in other pathways (**Figures 3B, C**). About 42 genes were related to carbohydrate metabolism, 33 genes were related to sugar metabolism, and 9 genes were related to fermentation. The 72 differential genes related to plant hormones were screened out, with genes related to auxin, CK, GA, abscisic acid (ABA) and strigolactone (SL) showing significant changes. Among them, 15 genes were related to auxin, 12 to CK, 19 to GA, 8 to ABA, and 6 to SL. There were a total of 12 genes related to brassinosteroid (BR), ethylene (ETH), and jasmonic acid (JA) metabolism (**Figure 3D**). Collectively, significant changes have taken place in the levels of transcripts related to lipid metabolism, carbohydrate metabolism and hormone metabolism, suggesting that AMF affected the regulation of these genes.

In shoots, of the 68 differential genes, 48 genes were related to primary metabolites (32 genes were related to lipid metabolism, 11 genes to carbohydrate metabolism, and 5 genes to amino acid metabolism) and 20 genes were related to secondary metabolites. Of the 32 genes related to lipid metabolism, 29 genes were down-regulated, and only 4 were up-regulated (related to fatty acid metabolism). Genes related to glycerolipid metabolism (11), phytosterol metabolism (3), and lipid trafficking (2) were all down-regulated (**Figures 3E, F**). The 42 differential genes related to hormone action were screened out, with genes related to JA (20), GA (7), ABA (5), CTK (3) and BR (3) showing significant changes (**Figure 3G**). Of the 20 genes related to JA, the 8 genes related to JA biosynthesis were down-regulated, and the 7 genes related to JA conjugation and degradation were also down-regulated. This may explain why there was no significant difference in the JA content between the inoculated and uninoculated plants, with only a 1.5-fold down-regulation seen with inoculation (**Supplemental Figure 5**). These data indicated that AMF might affect the expression and/or turnover of genes related to lipid and hormone metabolism.

### Carbohydrate metabolism and sugar accumulation in roots of apple plants in symbiosis with AM

**Figure 4:**
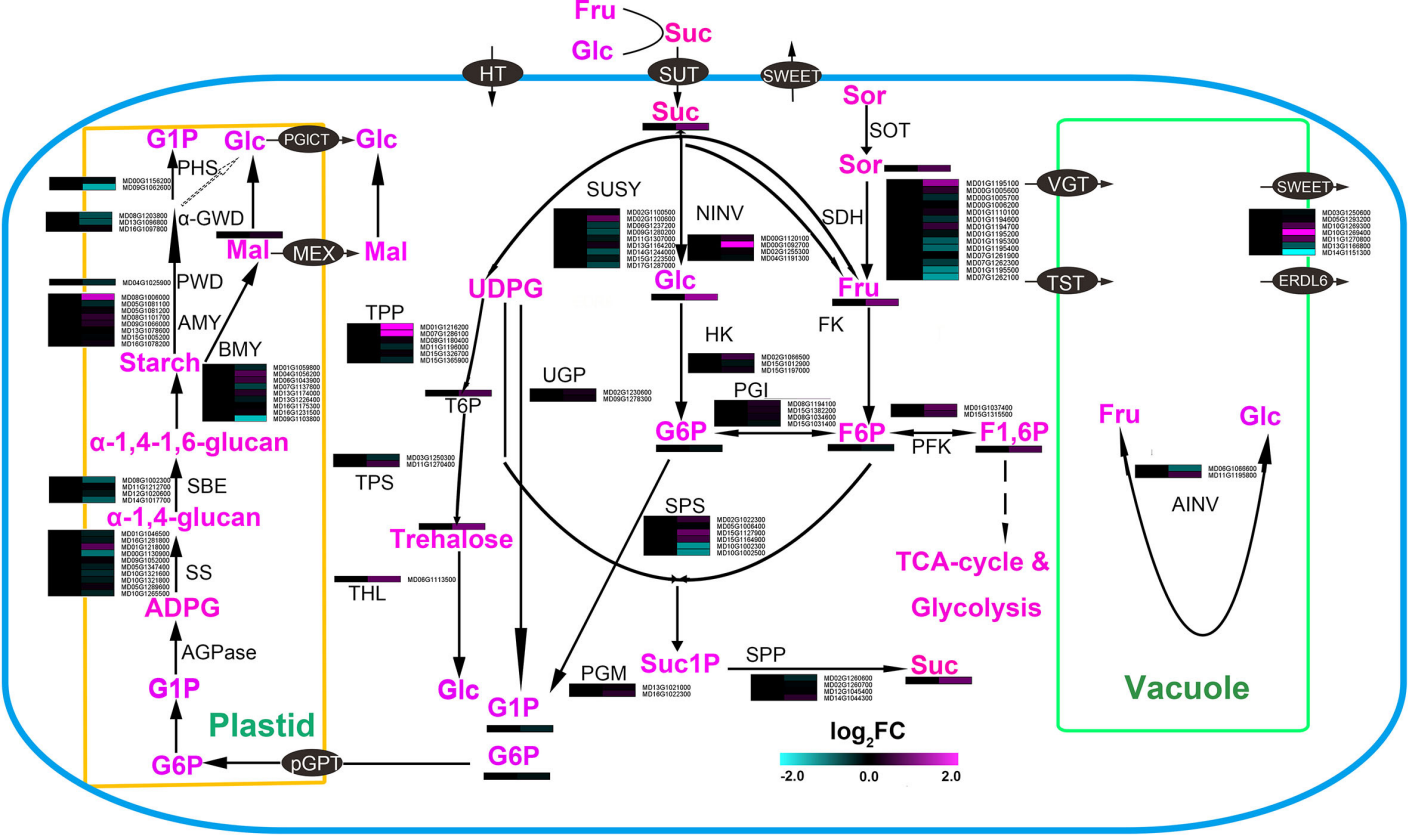
Main carbohydrate metabolism and sugar accumulation pathways in roots of apple plants inoculated with *R*. *irregularis* for 60 days. CWINV, cell wall invertase; FK, fructokinase; F6P, fructose-6-phosphate; Fru, D-fructose; Glc, D-glucose; G6P, glucose-6-phosphate; Tre, trehalose; Gal, galactose; GPT, glucose-6-phosphatetransporter; SDH, sorbitol dehydrogenese; HK, hexokinase; NINV, neutral invertase; PFK, phosphofructokinase; PGI, phosphoglucoisomerase; pGLCT, plastidic Glc transporter;PGM, phosphoglucomutase; SPP, sucrose-phosphate phosphatase; SPS, sucrose-phosphate synthase; SUSY, sucrose synthase; vAINV, vacuolar acidinvertase; vGT, vacuole glucose transporter; UGP, UDPG-pyrophosphorylase; BMY, beta amylase; HGL, heteroglycan glucosidase; PHS, α-glucanphosphorylase; AMY, α- amylase; α-GWD, α-glucan water dikinase; PWD, phosphoglucan waterdikinase; SBE, starch branching enzyme; SS, starchsynthase; TST, tonoplast sugar transporter; TPS, trehalose-phosphate synthase; α-gal, α-galactosidase; GALK, galactokinase; THL, trehalase; T6P, trehalose-6-phosphate.

The results of the joint metabolomic and transcriptomic analyses showed that sugar, hormone, and fatty acid pathways undergo significant changes when inoculated with AMF. Sugars, hormones, and fatty acids have been reported to influence root development (Trinh et al., 2019; Yamauchi et al., 2019; Valifard et al., 2021). To further explore how AMF changes of sugar, fatty acid and hormone contents, the sugar, fatty acid, and hormone metabolic pathways were mapped and integrated with the transcriptome and metabolome data (**Figure 4**).

Among the sugars, the relative contents of glucose (Glc), fructose (Fru), sucrose (Suc), trehalose (Tre) and sorbitol (Sor) increased over 50% after inoculating AMF. The largest change in relative content was for Glc. This accumulation of Glc might be caused through modification of two pathways (**Figure 4**): the increased relative content of trehalose and the increased relative expression of *neutral invertase* (*NINV*: *MD00G1092700*). The content of Suc was increased 90% (**Supplemental Figure 3**), although the transcripts for *sucrose phosphate synthase* (*SPS*) were not significantly up-regulated, there was a high, up-regulated relative level of expression of three *SPS1* genes *(MD02G1022300*, *MD15G1127900* and *MD15G116490*0). In addition, the content of Fru increased by 90% after inoculation with AMF (**Supplemental Figure 3**), the transcript levels of *sorbitol dehydrogenase* (*SDH*) showed a downward trend as a whole, and another *NINV* (*MD00G109270*) was significantly up-regulated. These data indicated that Fru may accumulate through an increased cleavage of Suc caused by AMF symbiosis.

### The responses of fatty acid synthesis and transport genes to AM symbiosis

The relative content of fatty acids showed an overall upward trend during AMF symbiosis. The relative expression levels of *acyl-ACP thioesterase A* (*FatA*) and *acyl-ACP thioesterase B* (*FatB*) showed no significant differences, but transcripts relevant to fatty acid synthesis, such as *ketoacyl-ACP synthase* (*KAS*: *MD06G1110900*, *MD14G1131500*, and *MD09G1022600*), were significantly up-regulated. The relative content of 14 diacylglycerol (DAG) was not significantly different, and the relative transcript levels of *phosphatase* (*PAP*) were little differences (**Supplemental Figure 4**).

### Synthesis and signal transduction of hormones in roots of apple plants in symbiosis with AM

**Figure 5:**
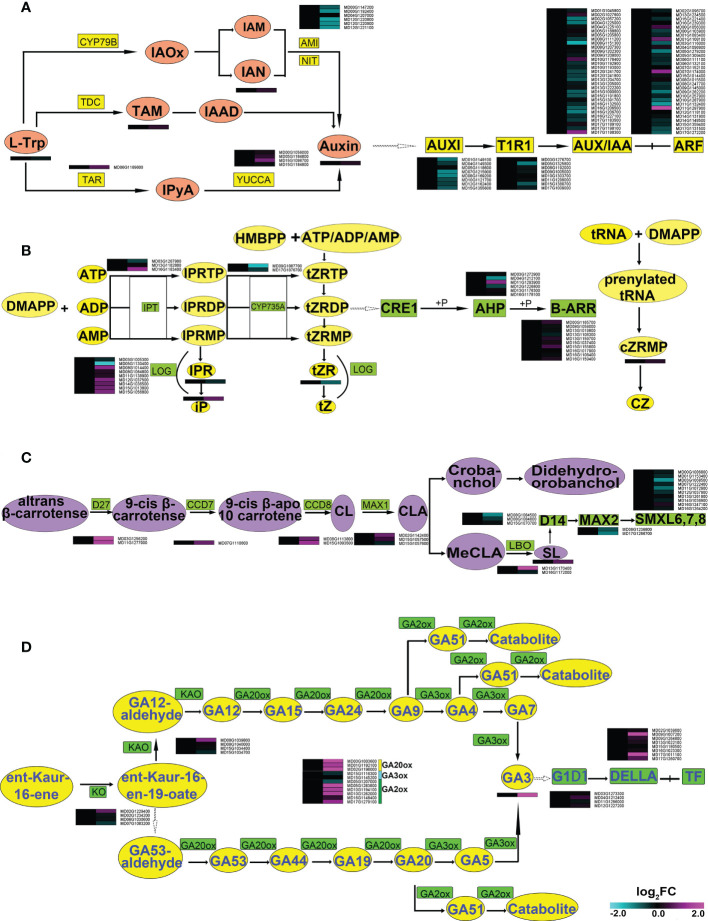
Synthesis and transduction pathways of hormones in roots of apple plants inoculated with *R*. *irregularis* for 60 days. **(A)** AMI1, AMIDASE 1; IAA, indole-3-acetic acid; IAAld, indole-3-acetaldehyde; IAM, indole-3-acetamide; IAN, indole-3-acetonitrile; IAOx, indole-3-acetaldoxime; IPyA, indole-3-pyruvic acid; L-Trp, L-tryptophan; NIT, nitrilase; TAR, tryptophan aminotransfearse related; TDC, tryptophan decarboxylase; CYP79B; AUX/IAA, transcriptional regulator family protein; ARF, auxin response factors; TIR1, transport inhibitor response1; **(B)** CTK, cytokinin; CRFs, cytokinin responsive factors; AHK3 and AHK4/CRE1, cytokinin response 1, 2; AHK2, Arabidopsis histidine kinase 2; type-B ARRs, Arabidopsis response regulators; AHPs, Arabidopsis histidine phosphotransferase proteins; IPT, isopentenyltransferase; CYP735A, cytochrome P450 monooxygenase; tRNA-specific IPT, tRNA-specific isopentenyltransferase; LOG, Lonely guy; iPRTP, iPR trphosphate; iPRDP, iPR diphosphate; iPRMP, iPR monophosphate; iPR, iP riboside; iP, N6-(2-isopentenyl)adenine; tZRTP, tZR 5′-trphosphate; tZRDP, tZR 5′-diphosphate; tZRMP, tZR 5′-monophosphate; tZR, tZ riboside; tZ, trans-zeatin; cZRMP, cZR 5′-monophosphate; cZ, cis-zeatin; DMAPP, Dimethylallylpyrophosphate; ATP, Adenosine triphosphate; ADP, Adenosine diphosphate; AMP, Adenosine monophosphate; HMBPP, 4-hydroxy-3-methyl-2-(E)-butenyl diphosphate; **(C)** DWARF27, D27; CCD7, carotenoid cleavage dioxygenase 7; MAX1, more axillary growth1; CCD8, carotenoid cleavage dioxygenase 8; SL, 5-deoxylstrigol; CL, carlactone; CLA, carlactonic acid; MeCLA, methy1 carlactonoate; LBO, oxidoreductase; **(D)** GA, gibberellin; KAO, ent-kaurenoic acid oxidase; KO, ent-kaurene oxidase; GA20ox, GA 20-oxidase; GA3ox, GA 3-oxidase; GA2ox, GA 2-oxidase; GID1, gibberellin insensitive dwarf1; DELLA, Gibberellic acid insensitive.

The transcriptome and metabolome both showed significant changes in auxin, CTK, GA and SL pathways, all of which are closely related to root growth (Yaxley et al., 2001; Zhou et al., 2011; Guillotin et al., 2017; Yamauchi et al., 2019). The content of indole-3-acetic acid (IAA) was increased (**Supplemental Figure 5** and **Figure 5A**), which correlated with the increased levels of transcripts within the whole *YUCCA* pathway. Conversely, the genes in the indole-3-acetamide (IAM) pathway showed a downward expression trend as a whole. Almost all genes related to auxin signal transduction were down-regulated, with only *ARF18* (*MD07G1174000*) and *ARF2* (*MD11G1297900*) significantly up-regulated. These data suggested that symbiosis with AMF might mainly change the expression of genes in the *YUCCA* pathway, affecting the synthesis of auxin.

For CTK, the contents of N6-(2-isopentenyl) adenine (iP) and cZR (CZ) were increased, and trans-zeatin riboside (tZR) and iP riboside (IPR) were decreased in AMF-inoculated apple roots. The form of CTK studied in the root was tZR (Li et al., 2018). In the CTK synthetic pathway, a *cytochrome P450 monooxygenase* (*CYP735A*: *MD09G1087700*) and *a cytokinin phosphoribohydrolase* (Lonely guy, *LOG*: *MD05G1130400*) were significantly down-regulated. Within the CTK signal transduction pathway, almost all genes showed no significant differences, only two *Arabidopsis histidine phosphotransferase proteins* (*AHP*: *MD04G1212100*, *MD11G1293900*) were significantly changed (**Supplemental Figure 5** and **Figure 5B**). These data indicated that AMF may affect the synthesis of tZR through changing the expression of CTK biosynthesis genes (*MD09G1087700*, *MD05G1130400*) in root.

Strigolactone (SL) was also up-regulated. The SL synthesis pathway showed an overall upward trend in expression. Conversely, the genes related to the SL transduction pathway showed downward trends (**Supplemental Figure 5** and **Figure 5C**). These data indicated that AMF may affect the expression of genes related to both synthesis and signal transduction of SL in root.

The content of GA3 was also significantly increased with AMF symbiosis. Genes related to both GA synthesis and signal transduction pathways showed overall up-regulation. In particular, the relative transcript levels of *DELLA* (*MD09G1007200* and *MD17G1011100*) were up-regulated over 38 times after inoculation with AMF (**Supplemental Figure 5** and **Figure 5D**), suggesting that AMF has a great influence on the transcription level of *DELLA*.

### Combined analysis of root and shoot metabolomes in symbiosis with AM

The environmental effects on plant growth and development have been studied extensively in both the below and above-ground biomass (Niklas, 2005). We found that AMF symbiosis significantly increased plant height (**Figure 1**). Next, we explored whether this increased shoot growth was caused by root development by analyzing the root to shoot ratio of metabolites, as follows: (AMF R/S - CK R/S)/CK R/S [Changes in root to shoot ratio before and after treatment], (AMF -CK)/CK R [Changes in root before and after treatment], and (AMF -CK)/CK S [Changes in shoot before and after treatment] (**Table 1**). This analysis revealed that only 26.4% of metabolites has unaltered R/S ratios (-20% < (AMF R/S-CK R/S)/CK R/S < 20%), while 73.6% of metabolites showed large differences in their R/S ratio (AMF R/S-CK R/S)/CK R/S >20%; (AMF R/S-CK R/S)/CK R/S <-20%). Among the metabolites with significantly different R/S ratios, about 40.3% of metabolites showed corresponding opposite trends (R+ S- or R- S+). These data showed that the trends in roots and shoots were very different. Therefore, we speculated that the shoot growth was not only caused by root development, but may also be closely related to AMF. How AMF mediated shoot development was analyzed next (**Table 1** and **Supplemental Table 2**).

**Table 1:** Variation of the root to shoot ratios of various metabolites in apple plants inoculated with *R*. *irregularis* for 60 days.

COMPOUNDS	(AMF R/S-CK R/S)/CK R/S>20%		-20%<(AMF R/S-CK R/S)/CK R/S<20%		(AMF R/S-CK R/S)/CK R/S<-20%	
	R+S+ Or R-S-	R+S+ Or R-S+	R+S+ Or R-S-	R+S+ Or R-S+	R+S+ Or R-S-	R+S+ Or R-S+
Amino acids and derivatives	10	11	11	2	3	2
Phenolic acids	21	17	12	5	9	16
Nucleotides and derivatives	3	5	6	3	5	6
Flavonoids	20	15	17	1	6	6
Lignans and Coumarins	4	5	2	1	2	1
Saccharides and Alcohols	8	5	10	3	4	5
Others	3	4	3	2	0	5
Tannins	2	1	4	0	0	0
Alkaloids	6	4	9	0	1	4
Terpenoids	8	10	0	2	4	2
Organic Acids	4	8	9	1	7	2
Lipids	10	17	19	4	13	14
Hormone	5	46	7	0	1	1
Sum	104	148	109	24	55	64

Tips: relative content, R, root; S, shoot; -, the ratio is negative; +, the ratio is positive; (AMF R/S-CK R/S)/CK R/S >20%, (AMF R/S-CK R/S)/CK R/S <-20%, significant difference; -20% < (AMF R/S-CK R/S)/CK R/S < 20%, no significant difference; the substances accumulated by hormone properties are summarized as > 20% (R+S-/R-S+, the number was 36); The numbers in the table are the number of trends in the root-to-shoot ratio of each metabolites; This is a summary of variation range of root to shoot ratio of various metabolites after inoculation. For full details see **Supplemental Table 2** in supplemental information online.

### Phenotypic analysis of shoot cells in apple plants in symbiosis with AM for 60 days

Cell development is closely related to plant height (Fu et al., 2002), so we wanted to determine if AMF affected cell development by observing the anatomical structures in the shoots of mycorrhizal and non-mycorrhizal plants. The cross-sections of mycorrhizal plants were not significantly different from non-mycorrhizal plants. In longitudinal sections, the cortex cells were longer and the node spacing increased in shoots with AMF (**Supplemental Figure 6A**). To confirm this visual observation, we measured the diameter and length of parenchyma cells. There was not a significant difference in the diameter of parenchyma cells between mycorrhizal and non-mycorrhizal plants, while the length of parenchyma cells was increased by 90% with AM symbiosis, which was significantly longer than non-mycorrhizal plants (**Supplemental Figure 6B**). Statistics of the relative expression levels of genes related to cell development (47 related to cell wall, 4 cell respiration, 4 cytoskeleton organization, and 5 cell division). The genes were related to cell wall, accounting for 78.3%.

Polar cell expansion in different tissues is critical for the development and morphogenesis of plant organs (Fu et al., 2002). Statistics of the relative expression levels of *EXPA* genes showed the *EXPA8 (MD01G1166700* and *MD11G1054500*), *EXPA15* (*MD00G1125400*) and *EXPA2* (*MD03G1052400*) were significantly up-regulated with AMF symbiosis. Four genes related to fatty acyl omega-hydroxylase (*CYP*) were significantly down-regulated. These data indicated that the change in cell morphology may be caused by changes in cell wall synthesis (**Supplemental Figure 6C** and **Supplemental Data 3**).

### Synthesis and signal transduction of hormones in shoots of apple plants in symbiosis with *R*. *irregularis*

**Figure 6:**
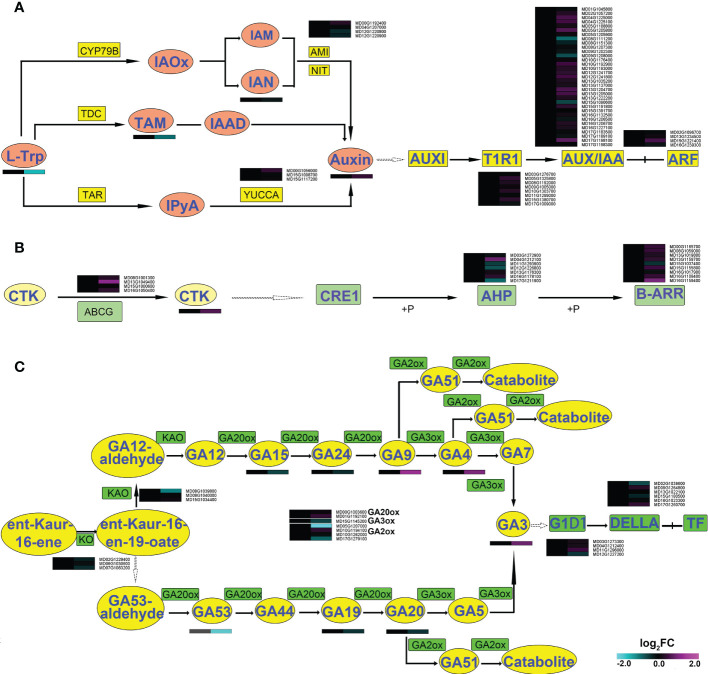
Synthesis, degradation, and transduction pathways of hormones in shoots of apple plants inoculated with *R*. *irregularis* for 60 days. **(A)** AMI1, amidase1; IAA, indole-3-acetic acid; IAAld, indole-3-acetaldehyde; IAM, indole-3-acetamide; IAN, indole-3-acetonitrile; IAOx, indole-3-acetaldoxime; IPyA, indole-3-pyruvic acid; L-Trp, L-tryptophan; NIT, nitrilase; TAR, tryptophan aminotransferase-related; TDC, tryptophan decarboxylase; TIR1, transport inhibitor response1; AUX/IAA, transcriptional regulator family protein; ARF, auxin response factors; **(B)** CTK, Cytokinin; ABCG, ABC transporter G family member; type-B ARRs, Arabidopsis response regulators; AHPs, Arabidopsis histidine phosphotransferase proteins; **(C)** GA, gibberellin; KAO, ent-kaurenoic acid oxidase; KO, ent-kaurene oxidase; GA20ox, GA 20-oxidase; GA3ox, GA 3-oxidase; GA2ox, GA 2-oxidase; gibberellin insensitive dwarf1, GID1; Gibberellic acid insensitive, DELLA.

Combined analysis of the transcriptome and the metabolome showed that there were significant changes related to auxin, CTK and GA, all of which influence shoot development (Ma et al., 2016; Zheng et al., 2018; Zheng et al., 2022). Significant changes related to auxin occurred at the metabolic level. For instance, the content of L-tryptophan (TRP) decreased 63.5%, while indole-3-acetic acid (IAA) increased 34.5% and N-(3-indolylacetyl)-L-valine (IAA-Val), 3-indoleacetonitrile (IAN), and indole-3-acetyl glutamic acid (IAA-Glu) increased over 2.5-fold (**Supplemental Figure 7**, **Figure 2** and **Figure 6A**). However, the transcriptome showed no significant changes in genes related to synthesis or signal transduction of auxin.

The content of the CTK trans-Zeatin riboside (tZR) increased 56.9% in the shoot of AMF-inoculated apple plants compared to the uninoculated mycorrhizal control plants (**Supplemental Figure 7**). The relative expression of the ABC transporter G family member (*ABCG*: *MD13G1049400*) was significantly up-regulated. In the CTK signal transduction pathway, the *AHP* was down-regulated, but the *B-type ARR* (*B-ARR*) was up-regulated (**Figure 6B**). Collectively, the AMF might affect expression of genes related to the transport and transduction of CTK, thereby causing a change in the CTK content.

The content of GA3 increased 87.7% in shoot of apple plants after inoculation with AMF (**Supplemental Figure 7**). The relative expression levels of almost all genes within the GA synthetic pathway, were decreased, especially *GA2-oxidase* (*GA2ox1*: *MD05G1207000*), which was significantly down-regulated. Genes in the GA transduction pathway did not show any significant differences in their transcript levels (**Figure 6C**). These data revealed that AMF may influence the content of GA3 by affecting its synthesis and degradation.

In short, the combined analysis of the differential metabolites and differentially expressed genes revealed a significant link between the transcriptomic and metabolomic signatures in apple plants in symbiosis with the AM *R. irregularis*. These results suggested that the metabolic signature of apple plants was transcriptionally controlled by inoculation with AMF. We used the metabolic and transcriptomic signatures to identify deregulated metabolic pathways. This analysis suggested that constitutive deregulation of sugar metabolism, hormone metabolism, and fatty acid metabolism in the roots of inoculated apple plants affected root growth and that the constitutive deregulation of hormone metabolism in the shoots affected shoot growth after inoculation with AMF. Cell development may also play an important role in response of shoot growth to symbiosis. According to the ratio (root/shoot) of metabolites, the increased shoot growth was a result of not only root development, but also the AMF symbiosis.

## Discussion

AMF promotes plant development and growth and can increase the tolerance to stress (Huang et al., 2020; Gao et al., 2020). However, it remains unknown how symbiosis with AMF precisely activates growth. In apple, stress resistance was improved after inoculation with AMF, which also promoted root and shoot development (Huang et al., 2020; Gao et al., 2020). The promotion of shoot and root development after AMF infection was also confirmed in our study (**Figure 1**). Exploring the molecular mechanism underlying the influence of AMF on plant growth and development will answer many questions about the symbiotic relationship. To determine how AMF promotes both shoot growth and root development, a combined analysis of both the root and shoot of uninoculated and AMF-inoculated apple plants was conducted through transcriptomics and metabolomics.

The trends of the metabolome and the transcriptome were basically compatible with each other, showing that AMF promotes plant development at both levels (**Figure 2** and **Figure 3**). We speculated that AMF mediates changes through the transcriptional control of metabolic pathways. In apple roots, we found that AMF regulation of development may involve multiple pathways, such as sugar, fatty acid, organic acid, and hormone metabolism (**Figure 2**), all of which may induce root development.

After AMF infection, a large amount of sugars usually accumulate in the root (Beltrano et al., 2013) and **Figure 4** and **Supplemental Figure 3**. At first, most researchers thought that sugars might be the carbon source for the AMF. The fungal symbiotic can take up and use hexose within the root, so the fungus would regulate the accumulation of sugar in the root system to meet its own needs (Solaiman and Saito, 1997). However, Jiang et al. (2017) found that fatty acids from the plant are transferred to the AMF, then converted to Glc by gluconeogenesis in the fungus. Therefore, it remains controversial why AMF regulates the accumulation of sugars in roots. However, accumulation of sugars in roots can affect root development. The accumulation of Fru can repress primary root growth, but induce the development of primary lateral roots, while the accumulation of Suc and Glc promotes the growth of primary and lateral root (Valifard et al., 2021). In our study, Glc, Fru, and Suc were all increased after inoculation with AMF (**Figure 4** and **Supplemental Figure 3**). However, the number of roots increased but the average root length did not after inoculation with AMF (Liu et al., 2018), which is the same as our phenotype (**Figure 1**). This may be a result of coordination between Glc, Fru, and Suc. Therefore, the accumulation of carbohydrates in roots may be another way for AMF to affect plant root development. Our analysis indicated that AMF increased the accumulation of carbohydrates in roots by regulating carbohydrate synthesis and transport. In our results, AMF promoted the relative expression levels of the *alkaline/neutral invertases* (*NINVs*: *MD00G1092700*) and *Trehalase* (*THL: MD06G1113500*), which may increase the content of Glc. The relative expression level of the sugar transporter *SWEET* (*MD10G1269400*) was significantly increased (**Figure 4**), which may affect the accumulation of sugar (Valifard et al., 2021). Fatty acids, as the form of carbon transport from host plants to AMF, also over accumulated in the root system (Jiang et al., 2017; Wu et al., 2019). The content of total fatty acids in the root of mycorrhizae-associated jujube plants was significantly higher non-mycorrhizal jujube plants (Ma et al., 2022). In our results, the relative content of fatty acids (Cis-5,8,11,14,17-Eicosapentaenoic Acid, Arachidonic Acid, and 9-Hydroxy-13-oxo-10-octadecenoic Acid) was also significantly increased (**Figure 2**. and **Supplemental Figure 4**). Interestingly, fatty acids also significantly promote root development (Trinh et al., 2019). It seems that AMF regulates the accumulation of fatty acids in roots, which may be another way that AMF affects plant root development.

The relative content of all the differentially regulated organic acids were increased in roots inoculated with mycorrhizae (**Figure 2A**). Accumulated organic acids can increase the acidity of the rhizosphere (Harker et al., 2002). Acidic conditions are more favorable for apple root growth (Kochian et al., 2015). Although it is common that AMF promotes the accumulation of organic acids in plant roots, not all plant roots are adapted to acidic conditions, such as tomato, rice and barley (Liu et al., 2016; Cristina et al., 2020; Szurman-Zubrzycka et al., 2021). Hage-Ahmed et al. (2013) found that tomato inoculated *Glomus mosseae* (AMF) had increased levels of protocatechuic acid and salicylic acid, which is in line with our findings. Therefore, we think that the accumulation of organic acids in roots may be a factor of AMF promoting root development of plants adapted to acidic growth conditions, but it may not be applicable to all species.

An enrichment of genes related to the response to hormones indicated the altered hormonal responses in apple plants under inoculation (**Figure 3D**; **Supplemental Figure 5**, and **Supplemental Data 3**). Further analyses revealed that the IAA, CTK, GA and SL responses were altered. Symbiosis between AMF and plants is often accompanied by changes in hormone levels. Auxin is essential for root growth (Yamauchi et al., 2019). The biosynthesis of auxin was promoted following AMF inoculation (Kaldorf and Ludwig-Müller, 2000). In our results, the content of IAA was increased (**Figure 5A** and **Supplemental Figure 5**). A previous study showed that *YUCCA* can convert tryptamine (TAM) to N-hydroxytryptamine (NHT) *in vitro* (Zhao et al., 2001), suggesting that *YUCCA* catalyzes the N-oxygenation of TAM, a rate-limiting step in auxin biosynthesis in many plants. The relative expression levels of three *YUCCA* genes (*MD15G1098700*, *MD00G1056000* and *MD05G1184800*), *ARF18* (*MD07G1174000*) and *ARF2* (*MD11G1297900*) were upregulated (**Figure 5A**). This may induce the increased IAA content, which would promote root development.

Likewise, CTK also affects root development, as a higher ratio of AUX/CTK promotes DEFINE (LR) formation (Zhou et al., 2011). In our result, the relative expression of the CTK synthetic gene *CYP735A* (*MD09G1087700*) was significantly reduced, which may reduce the content of CTK (tZR) (**Figure 5B**). The increased IAA and decreased tZR would alter the AUX/CTK ratio of mycorrhizae-associated apple plants, which was higher than the uninoculated apple plants. In summary, the changes in the AUX and CTK contents due to AMF symbiosis are beneficial to root development.

A previous study showed that the content of SL was increased after inoculation with AMF (Guillotin et al., 2017), which is consistent with our results. The relative expression of SL synthesis genes, such as *D27* (*MD03G1256200*, *MD11G1277000*), *CCD8* (*MD15G1093500*) and *LBO* (*MD16G11720000*), were significantly up-regulated, but the relative expression levels of SL signal transduction genes *D14* (*MD08G1084500*) and *SMXL6* (*MD03G1068500*) were significantly down-regulated (**Figure 5C**). SL promotes primary root development, while the analysis of mutants in SL synthesis or signaling suggest that the absence of SL enhances lateral root formation (Kapulnik et al., 2011; Kumar et al., 2015). These results indicated the AMF may inhibit SL signal transduction to promote root development.

The growth of the aerial parts of the plant is closely related to the growth condition of the underground parts (Ko and Helariutta, 2017). In our study, the ratio of the aerial to underground parts of apple plants did not change significantly before and after AMF infection (**Figure 1K**). Therefore, the promotion of root development by AMF is likely a key factor affecting shoot growth. On the other hand, a comprehensive analysis of the transcriptomes and metabolomes of roots and shoots showed that both primary and secondary metabolites were quite different between the shoots and roots. The most drastic change was the 38 metabolites that specifically accumulated in the shoot. In the shoots, there were 17 metabolites that specifically accumulated with AMF inoculation, and there were 21 metabolites specific to the uninoculated plants, but no metabolites were specific to the inoculated roots (**Table 1** and **Supplemental Table 1**). Comparing the anatomical structure of the shoot of inoculated and uninoculated plants showed that the shoot cells were significantly longer after inoculation (**Supplemental Figure 6A**). Therefore, we have full reason to speculate that the promotion of shoot growth by AMF was not merely due to better root development, as AMF also directly regulates shoot growth and development.

AMF clearly regulates aerial growth and development, as the plants grew significantly taller after AMF inoculation (**Figure 1B**) and AMF regulates some metabolites and genes related to plant growth, such as hormone (IAA, CTK, and GA) synthesis and signaling and cell morphogenesis. IAA, CTK, and GA promote shoot development (Ma et al., 2016; Zheng et al., 2018; Zheng et al., 2022), and AMF promotes stem growth by involving IAA, CTK, GA and other hormones (Dugassa et al., 1996; Barker and Tagu, 2000). Our combined shoot transcriptome and metabolome indicates that AMF regulates hormone synthesis, by affecting transcription related to hormone synthesis in the shoot, such as the genes *YUCCA10* (*MD00G1056000*) and *GA2ox* (*MD05G1207000*) (**Figure 6A, C**), and hormone signal transduction, such as the genes *ARF19* (*MD15G1221400*) and *B-ARR* (*MD16G1159400*) (**Figures 6A, B**). The cell morphology of shoot tissue was significantly changed after inoculation with AMF (**Supplemental Figures 6A, B**) and the transcriptome showed that genes related to cell morphogenesis were significantly changed (**Supplemental Figures 6C**). We hypothesized that AMF regulates plant growth by affecting the transcription of morphogenesis-related genes.

Sugar did not seem to play an important role in shoot growth, as neither the metabolites nor the genes changed a lot (**Figure 2** and **Figure 3**). Eighty-two flavonoids showed large changes in response to AMF inoculation (**Figure 2**, **Figure 3**; **Supplemental Data 2**, and **Supplemental Data 3**), possibly triggered by *MYC2* in a JA- and ABA-dependent manner (Adolfsson et al., 2017). It is unknown what roles this change might play in shoot growth in plants in symbiosis with AMF; this might be a meaningful scientific question to study.

Although AMF promoted the growth and development of plant roots and shoots, the colonization rate of AMF varies greatly among species under natural conditions. For instance, the natural colonization rate of soybean was around 40%, and that of wheat was lower than 20% (Gao et al., 2012; Brito et al., 2012). The colonization rate of AMF also was influenced by external environmental factors such as climate, soil, and management patterns (Gosling et al., 2006; Gosling et al., 2010). A high colonization rate is essential for AMF to promote the plants growth and development, and two years of organic management significantly increased the colonization rate of AMF on onions (Gosling et al., 2010), but less research has been studied on other species, so the widespread use of AMF in production still requires extensive field experiments to explore.

## Materials and methods

### Plant material and experimental treatments

This experiment was carried out in the Northwest A&F University Greenhouse of Shaanxi Province. Plants were derived from cuttings and were maintained in the greenhouse in plastic pots (21 cm ×15 cm) filled with sand (sterilized by steaming at 121°C for 2 h). There were a total of 200 plants of the rootstock M.26. Plants with a height of about 10 cm were selected, of which 100 plants were in the treatment group and 100 plants in the control group. The AMF was *Rhizophagus irregularis* (strain number: BGC BJ09). The inoculant contained about 60 spores per gram. The treatment group was inoculated with 20 g AMF inoculant, while the control group was inoculated with 20 g sterilized AMF. Place the plants in the greenhouse after treatment (the relative humidity was 55-75%, and the temperatures were 12-30 °C). The plants were watered normally, and all plants were watered with 100 mL ½-strength low-phosphorus Hoagland’s weekly. Sixty days after inoculation with AMF, the plants were measured, and samples were collected.

### Mycorrhizal colonization and morphological indices assays

The method used for mycorrhizal colonization observation was described by He et al. (2016), with minor modifications. As follows, the roots were cleaned and then soaked in 10% KOH until transparent and softened. Stained with 0.05% trypan blue, and decolorized with 1:1 actic acid-glycerin for 3 times. Final compression and microscopic examination are performed. At the end of the 60-day co-culture period, plant height, internode length, internode number, root number, and root length were measured. We used an EPSON EXPRESSION 10000XL scanner (LAl600 scanner, Canada) to scan the samples and obtain the total root length. As for the number of roots, we performed a manual count. The net photosynthetic rate was measured by Li6800 (LICOR, Huntington Beach, CA, USA). Selected fully developed mature leaves at the same node to measure net photosynthetic rate, and at least 9 plants were as one biological replicate. At the same time, the chlorophyll content was measured by the acetone method (Chazaux et al., 2022), with the leaves in the same position were selected for determination. The rhizome ratio was calculated using the ratio of root to shoot.

### Transcriptome analysis of apple plants in root and shoot

The root tips and the shoot tips were used to extract the RNA, with the protocol of *kit (TIANGEN). All primers used are shown in **Supplemental Table 1**. A high-quality cDNA library was constructed using. Transcriptome sequence was performed on an Illumina HiSeq platform (Illumina, San Diego, CA, USA). Raw data onto the fast format (original reads) were processed by internal perl script. After program processing, high quality reads were obtained. High-quality reads were mapped to the reference genome (https://www.rosaceae.org/species/malus/all), and the FPKM value of each gene was calculated. Using PCA to assess the relationships of samples. Differential genes were screened on P value <0.05 and fold change ≥2.0. The KO (KEGG orthogonal) and MapMan databases were used to annotate the functions of DEGs.

### Metabolome analysis of apple plants in root and shoot

Untargeted metabolomics were used to differentially identify the primary metabolites and secondary metabolites by UPLC - MS/MS analysis. Using PCA to assess the relationships of samples. Differential metabolites were screened on VIP ≥1.0 and fold change ≥2.0. Add 0.1g of sample to 1.2ml of 70% methanol, vortex and shake to mix well. The UPLC system was Thermo Vanquish and column was Accucore Vanquish (Agilent SB-C18 1.8 µm, 2.1 mm * 100 mm). The program was set as follows, with a flow rate of 0.35 mL/min, a column temperature of 40°C, and an injection volume of 4 µL each time. Solvent A was water/0.1% formic acid and solvent B was ACN/0.1% formic acid. For mass spectrometry (MS) analysis, the setup procedure was as follows: the electrospray ionization (ESI) temperature was 550°C, and the GSI, GSIII and curtain gas (CUR) pressures were 50 psi, 60 psi and 25 psi, respectively.

Hormone metabolomics were used to identify differential hormones by LC-MS/MS analysis. Using PCA to assess the relationships of samples. Differential hormone metabolites were screened on P value <0.05 and fold change ≥2.0. The 0.05 g of samples, 10 µl of a mixed internal standard solution (10 ng/ml) and 1 ml methanol/water/formic acid (15:4:1, V/V/V) by vortex to mix well. The UPLC system was Thermo Vanquish and column was Waters ACQUITY UPLC HSS T3 C18 (1.8 µm, 100 mm×2.1 mm i.d.). The program was set as follows, with a flow rate of 0.35 mL/min, a column temperature of 40°C, and an injection volume of 4 µL each time. Solvents A and B were water/0.04% acetic acid and ACN/0.04% acetic acid, respectively. For mass spectrometry (MS) analysis, the Electrospray Ionization (ESI) temperature was 550°C, the Curtain Gas (CUR) pressure was 35 psi.

## Statistical analysis of data

Data analysis with SPSS 22.0, the significant differences were determined using Duncan’s test in a one-way ANOVA (p < 0.05).

## Data availability statement

The data presented in the study are deposited in the NCBI repository, accession number PRJNA889983.

## Author contributions

MZ, ML and FM: conceived and supervised this study; SJ, TY and BL: performed the experiments; YL, LZ and JS: performed the bioinformatics analysis; SJ: wrote the manuscript; ML, BM, MZ and SJ: discussed the study and revised the manuscript. The authors responsible for distribution of materials integral to the findings presented in this article in accordance with the policy described in the Instructions for Authors are MZ (mrz@nwsuaf.edu.cn), ML (limingjun@nwsuaf.edu.cn) and FM (fwm64@nwsuaf.edu.cn). All authors contributed to the article and approved the submitted version.

## Funding

This research was funded by the Apple Research System (CARS-27).

## Acknowledgments

We thank Dr Jing Zhang, Miss Jing Zhao, and Miss Beibei He (Horticulture Science Research Center, Northwest A&F University, Yangling, China) for providing professional technical assistance with UPLC–MS/MS analysis and Microscopy technique.

## Conflict of interest

The authors declare that the research was conducted in the absence of any commercial or financial relationships that could be construed as a potential conflict of interest.

## Publisher’s note

All claims expressed in this article are solely those of the authors and do not necessarily represent those of their affiliated organizations, or those of the publisher, the editors and the reviewers. Any product that may be evaluated in this article, or claim that may be made by its manufacturer, is not guaranteed or endorsed by the publisher.
