# Supplementary material for: Transcriptomics and metabolomics reveal effect of arbuscular mycorrhizal fungi on growth and development of apple plants
Source: Front Plant Sci. 2022 Oct 25;13:1052464. doi: 10.3389/fpls.2022.1052464 (PMC9641280; doi:10.3389/fpls.2022.1052464)
Supplement: Supplementary file 5 [file DataSheet_5.docx]

**
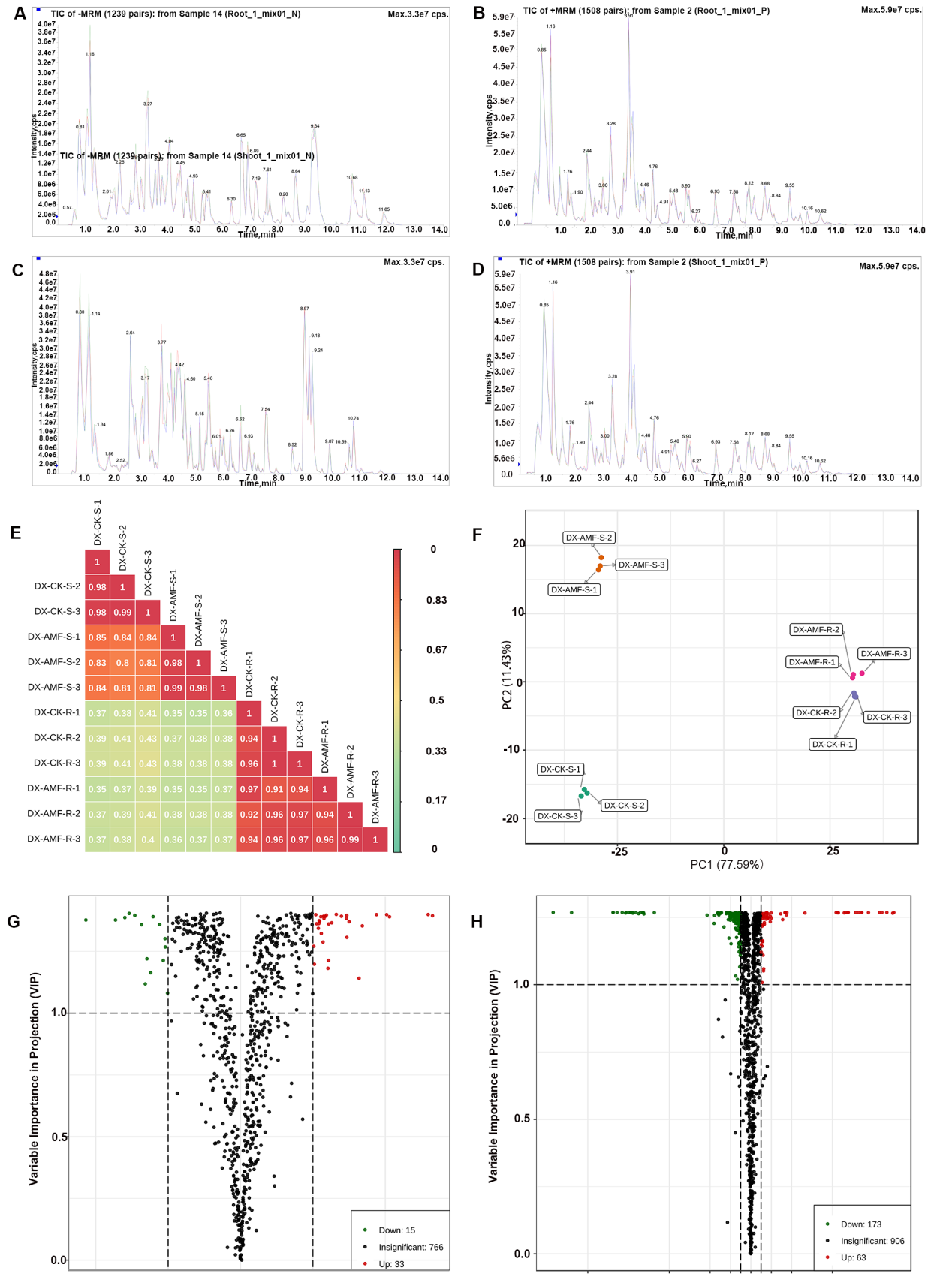
Supporting information**

**Supplementary Figure 1.** Quality control of the metabolomics analysis of 12 samples in root and shoot of apple plants incoculated with *R*. *irregularis* after 60 days. N stands for positive ion mode， and P stands for negative ion mode. (A, B) Overlapping chromatogram of mass spectrometry detection-N, P in root of apple plants. (C, D) Overlapping diagram mass spectrometry detection-N, P in shoot of apple plants. (E, F) Correlation analysis and Principal Component Analysis (PCA) of 12 samples of apple root and shoot. (G, H) Volcano plots of different metabolites in apple roots and shoots, comparing uninoculated and inoculated plants.

**
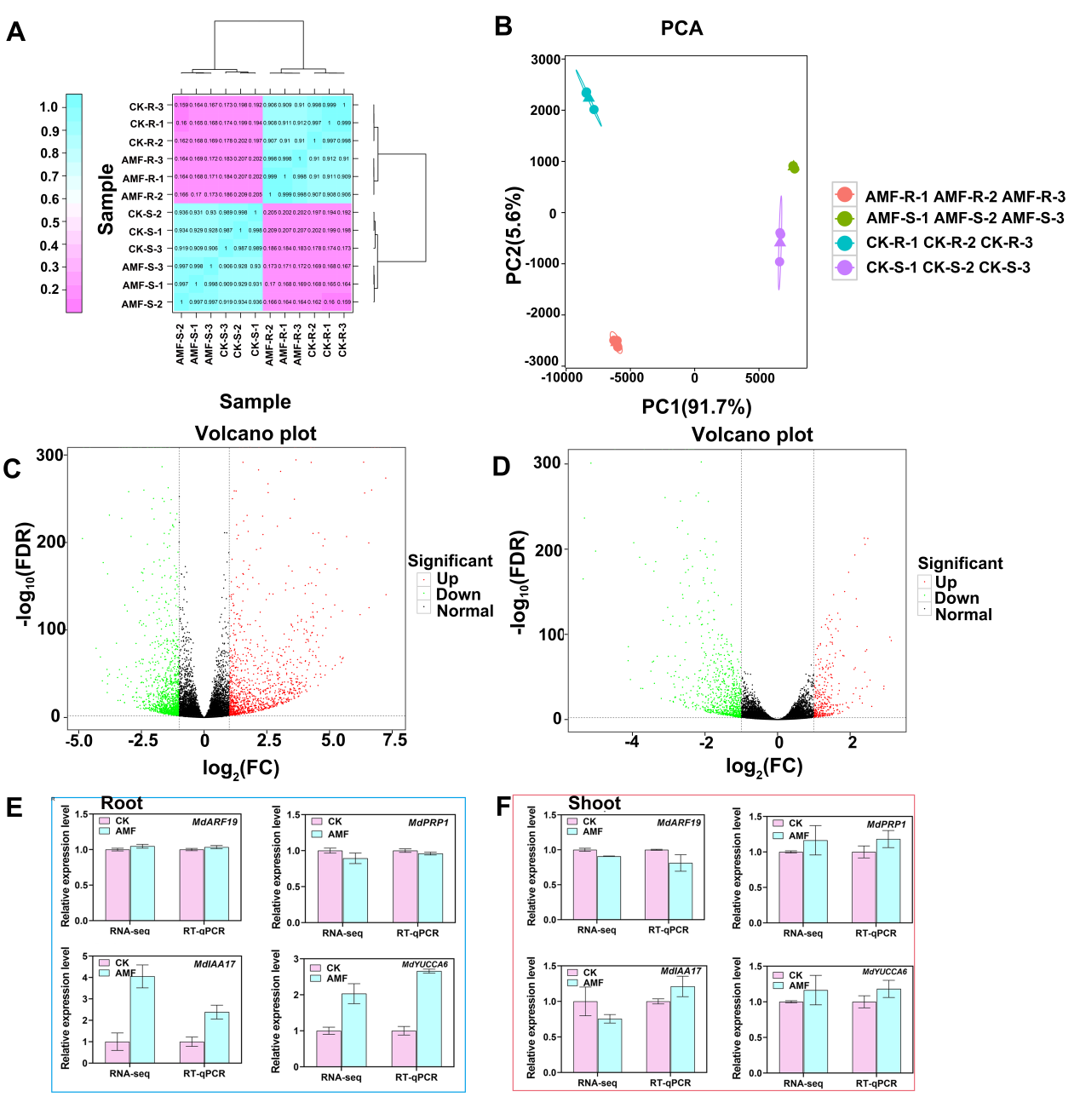
**

**Supplemental Figure 2**. Quality control of transcriptomics by RNA sequencing of 12 samples of roots or shoots from apple plants inoculated with *R*. *irregularis* or uninoculated after 60 days. Correlation analysis (A) and PCA analysis (B) of the 12 samples. Volcano plot of different metabolites in root (C) and shoot (D) samples. Relative expression levels of 4 genes in roots (E) and shoots (F) of apple plants by RNA-seq and RT-qPCR.

**
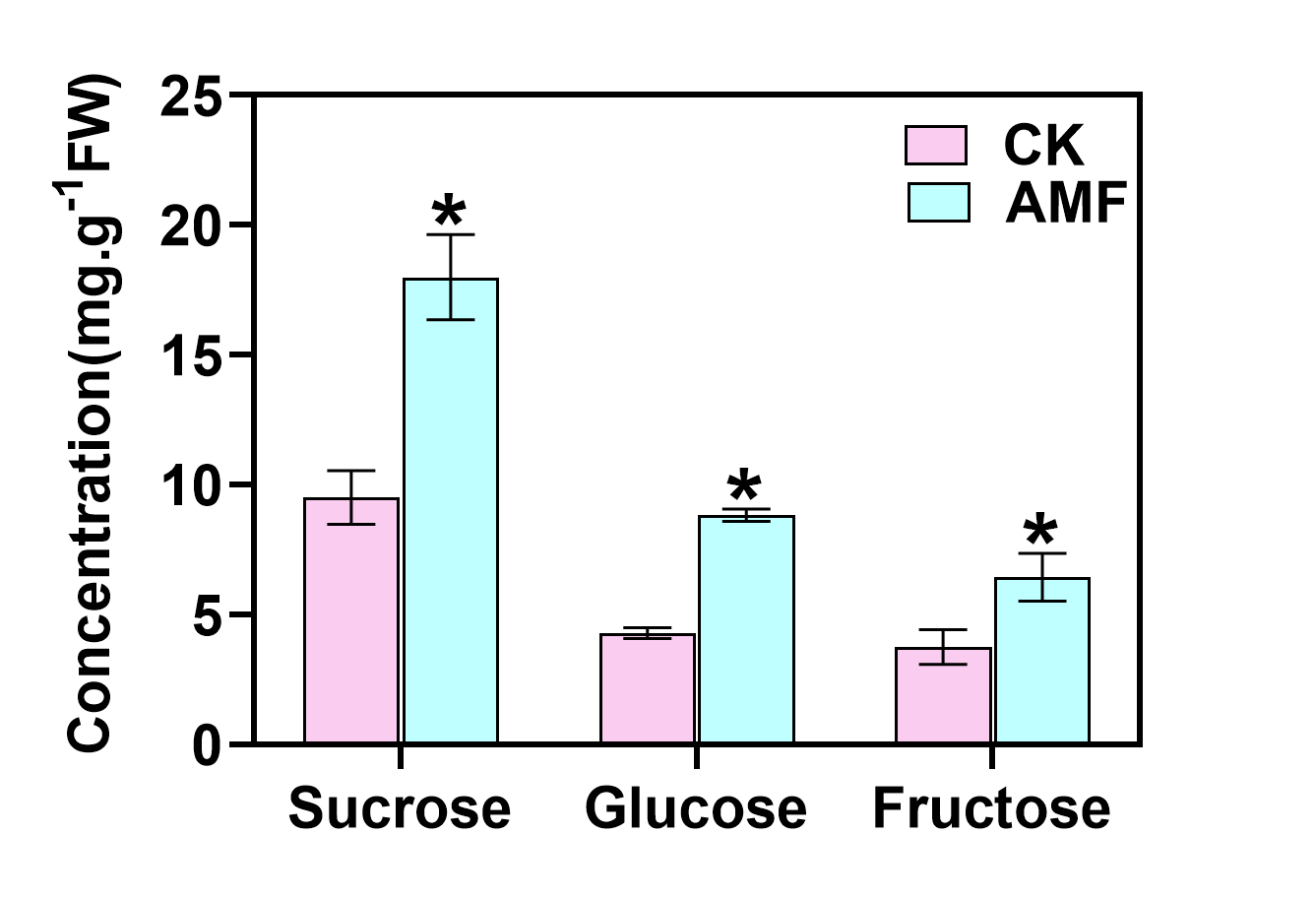
**

**Supplemental Figure 3**. The content of sugars in roots of apple plants inoculated with *R*. *irregularis* for 60 days. Values are the means ± SD of three biological replicates. * Indicated a significant difference at 0.05 level, ** Indicated a significant difference at 0.01 level.

**
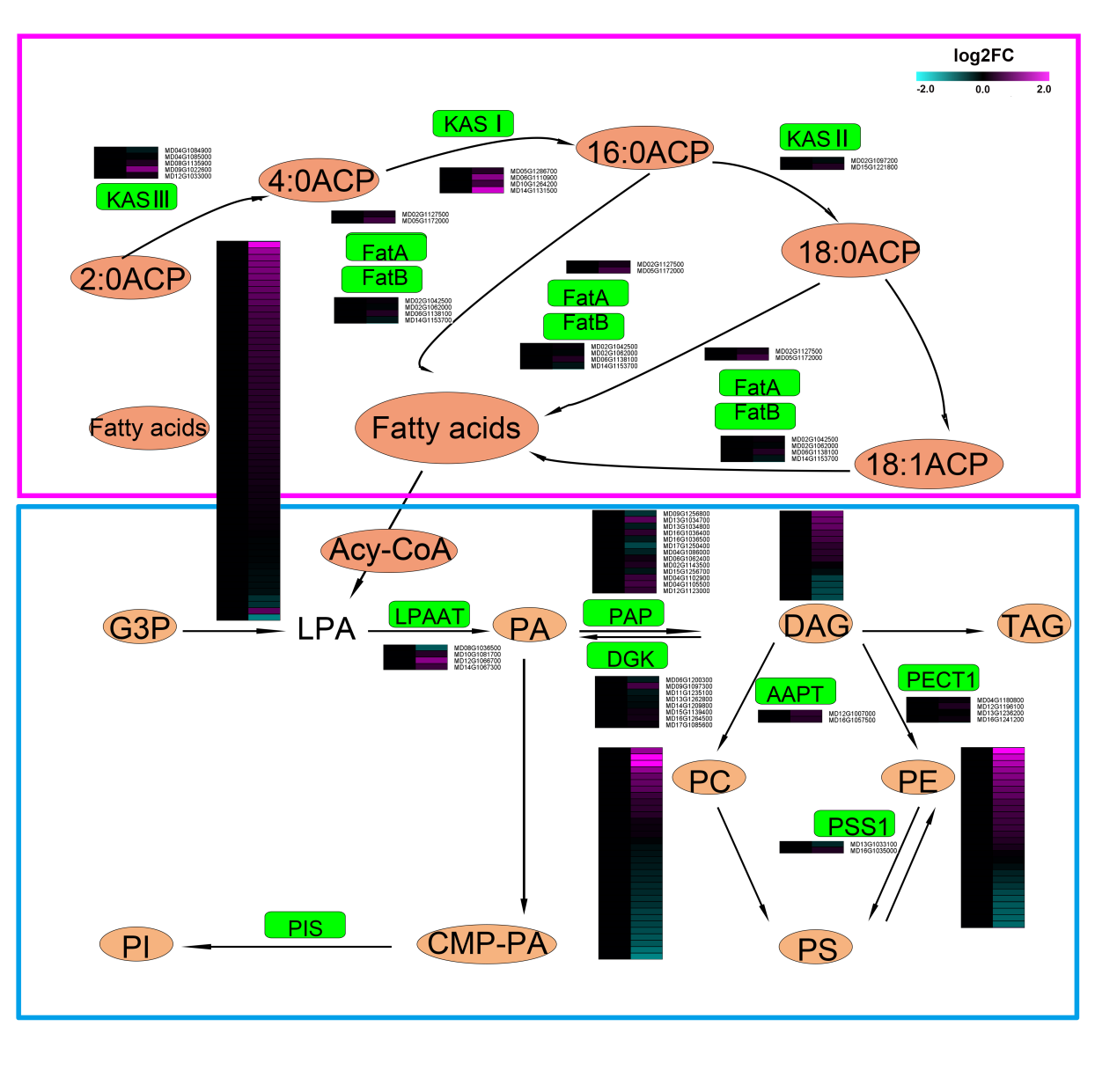
**

**Supplemental Figure 4.** Expression profiles of genes involved in fatty acid (FA) biosynthesis, processing, and transportation in roots of apple plants inoculated *R*. *irregularis* for 60 days. ACP, acyl carrier protein; KAS, ketoacyl-ACP synthase; FatA, acyl-ACP thioesterase A; FatB, acyl-ACP thioesterase B; DAG, diacylglycerol; PA, phosphatidic acid; PC, phosphatidylcholine; PE, phosphatidylethanolamine; PS, phosphatidylserine; PI, phosphatidylinositol; LPAAT, lysophosphatidic acid acyltransferase; PAP, phosphatase phosphatase; AAPT, aminoalcohol aminophosphotransferase; DGK, DAG kinase; PECT, cytidine triphosphate: phosphorylethanolamine cytidylyltransferase; PSS, PS SYNTHASE; CDS, CYTIDINE DIPHOSPHATE-DAG SYNTHASE; CMP-PA, CYTIDINE MONOPHOSPHATE PA; TAG, triacylglycerol.

**
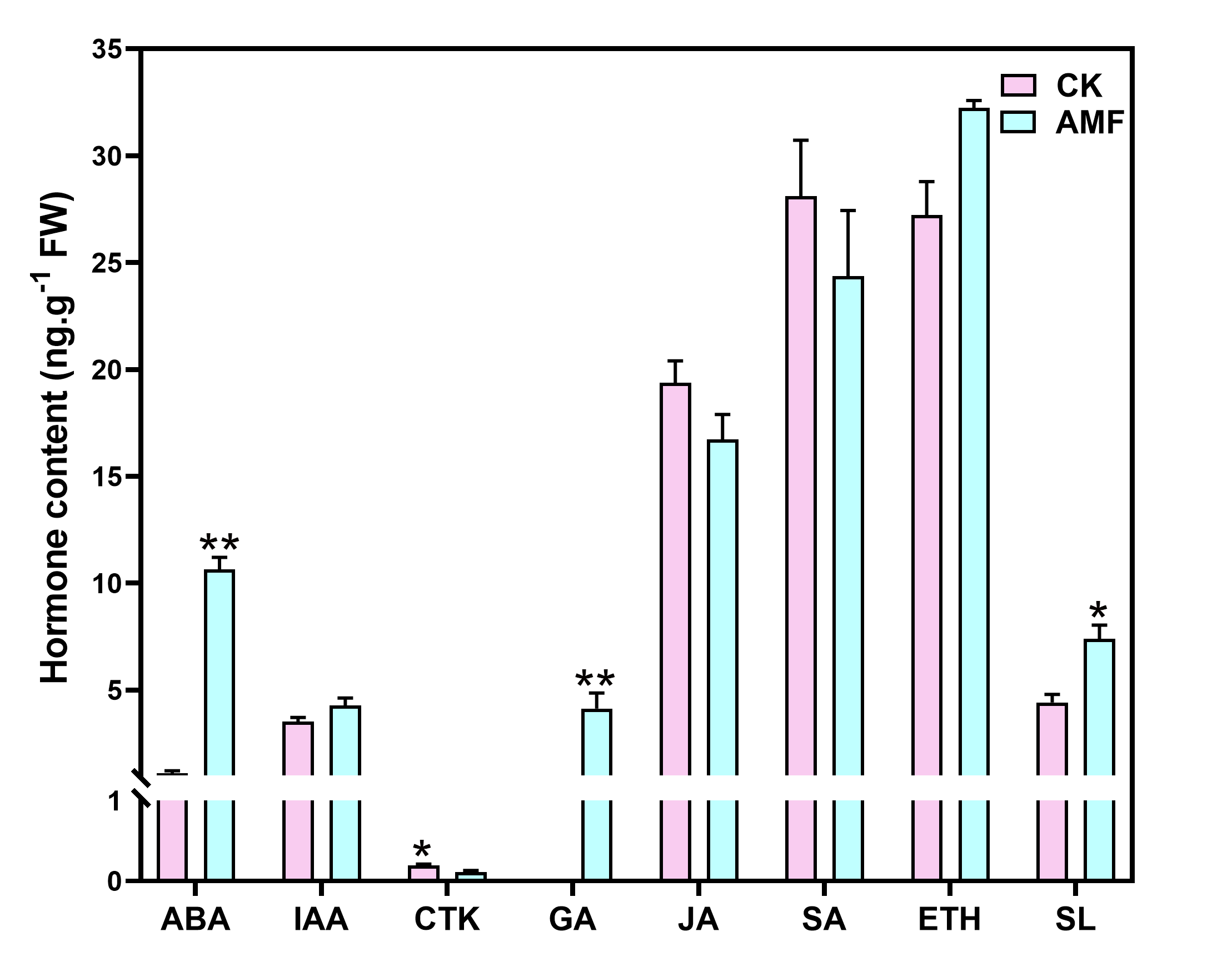
**

**Supplemental Figure 5.** The contents of hormones in roots of apple plants inoculated with *R*. *irregularis* for 60 days. Values are the means ± SD of three biological replicates. * Indicated a significant difference at 0.05 level, ** Indicated a significant difference at 0.01 level.

**
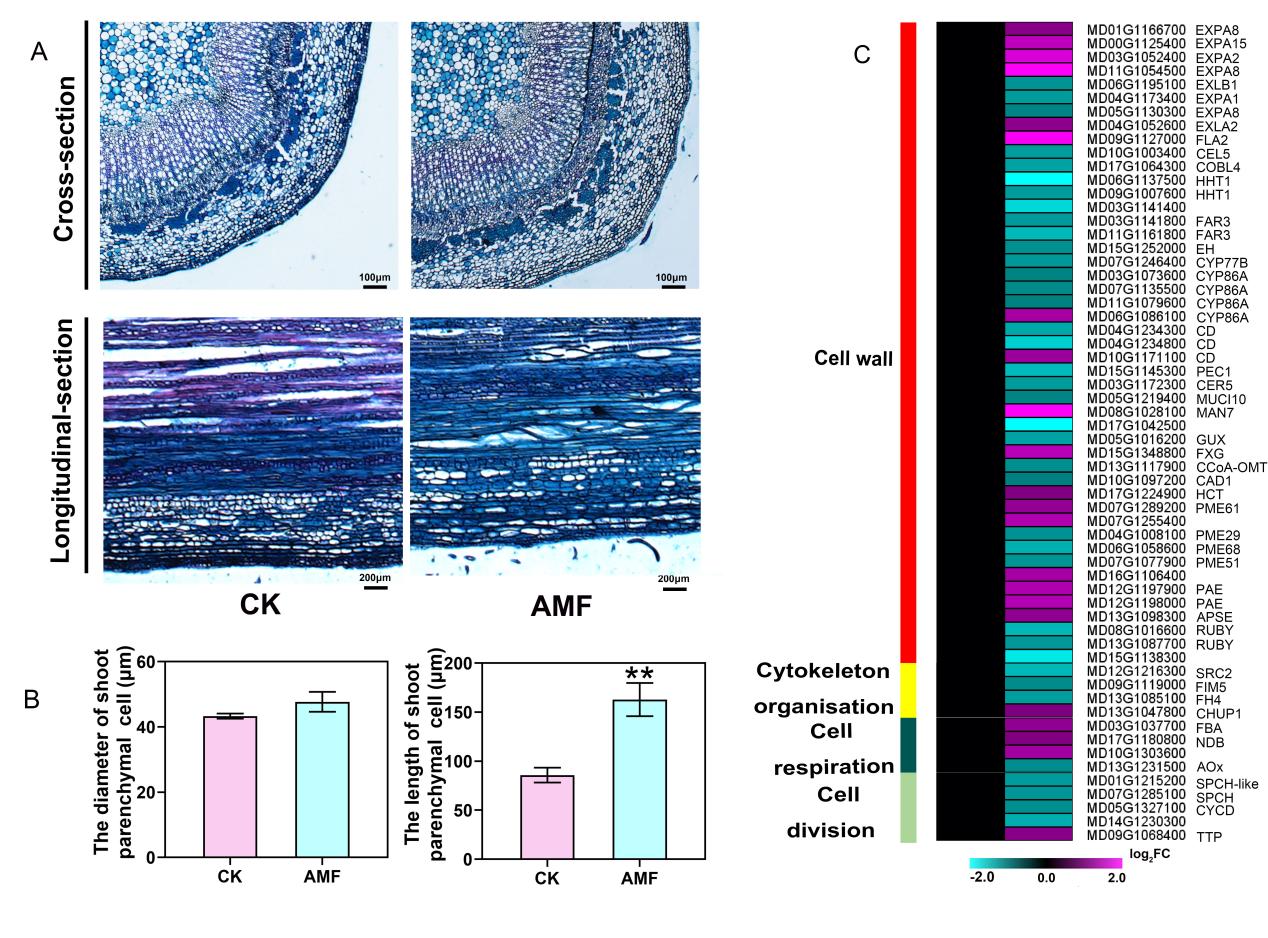
**

**Supplemental Figure 6. Anatomical structure of apple shoot and differentially expressed genes related to cell development.** (A) Cross-section and longitudinal section of shoots in AMF-inoculated apple plants and uninoculated apple plants (CK). (B) Diameter and length of parenchyma cells in AMF-inoculated apple plants and uninoculated apple plants (CK). (C) Differential expression of genes related to cell development.

**
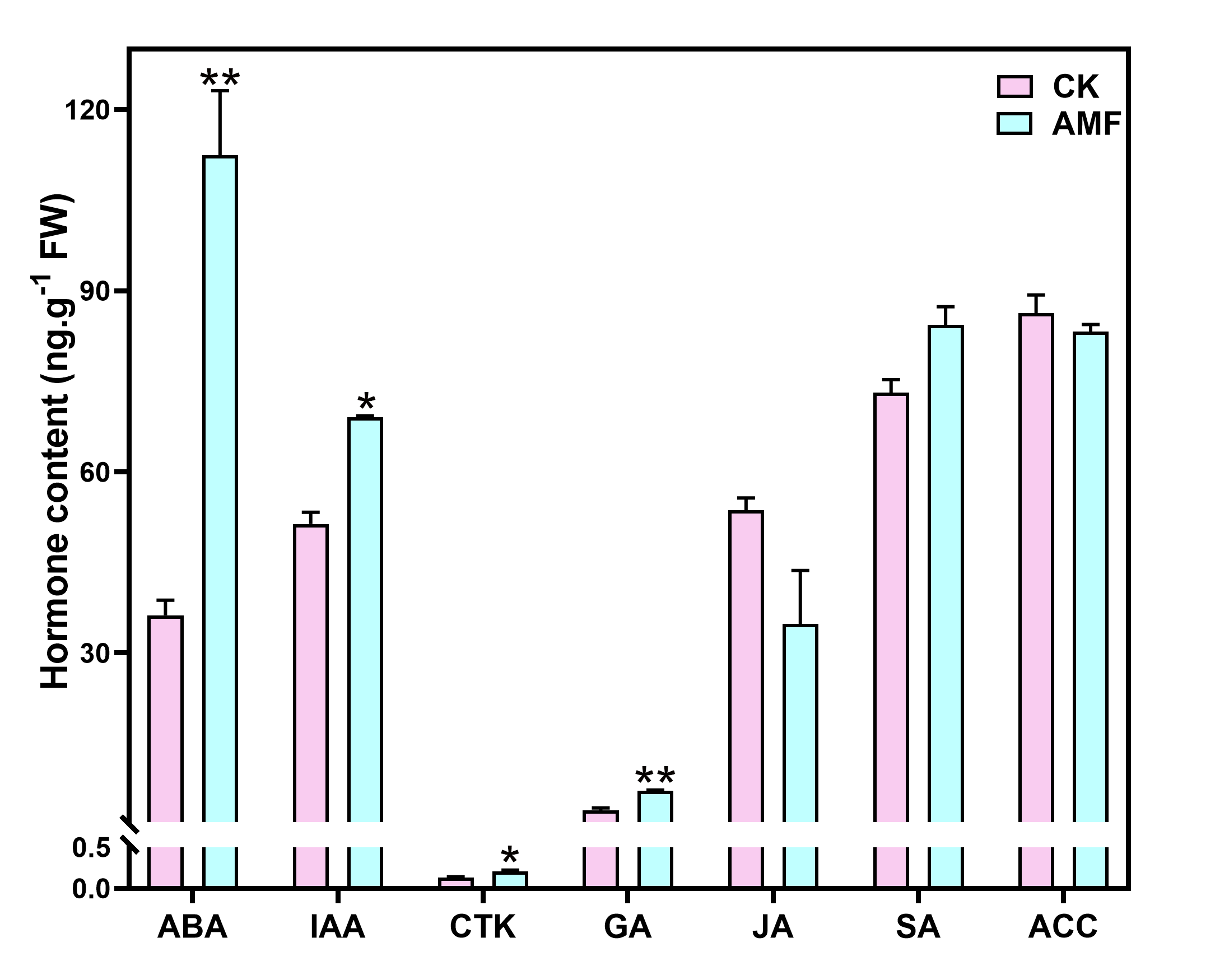
**

**Supplemental Figure 7.** The contents of hormones in the shoots of apple plants in symbiosis with *R*. *irregularis* for 60 days. Pink bars show hormone levels in uninoculated plants. Blue bars show hormone levels in inoculated plants. Values are the means ± SD of three biological replicates. * Indicate a significant difference at 0.05 level, ** Indicate a significant difference at 0.01 level.

[**Supplementary**](D:/Dict/8.10.3.0/resultui/html/index.html#/javascript:;) **table 1** The primers used for qRT-PCR


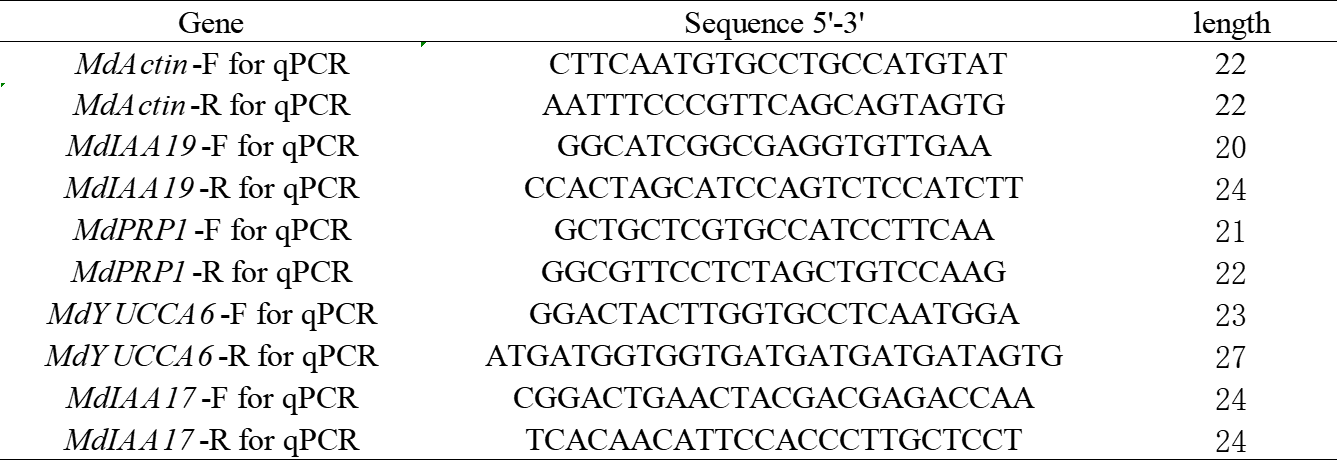


[**Supplementary**](D:/Dict/8.10.3.0/resultui/html/index.html#/javascript:;) **table 2** The root to shoot ratio of differential metabolites in apple plants inoculated with *R*. *irregularis* for 60 days
